# A dual mediation model of the association between principal inclusive leadership and teacher innovative behavior

**DOI:** 10.1038/s41598-024-63332-0

**Published:** 2024-05-29

**Authors:** Yuangen Bao

**Affiliations:** Department of Educational Research Management, Shenzhen Longgang Teacher Development Institute, Qinglin Avenue, Shenzhen, 518172 Guangdong People’s Republic of China

**Keywords:** Inclusive leadership, School innovation climate, Teacher psychological empowerment, Teacher innovative behavior, Cross-level analysis, Human behaviour, Environmental social sciences, Psychology and behaviour

## Abstract

Teacher innovation is crucial for the education system to adapt to contemporary society's evolving demands. However, the underlying mechanism of how inclusive leadership positively impacts employees’ innovative behavior remains incomplete. Therefore, drawing upon the social cognitive theory, this study aims to comprehensively examine the mediating effects of teachers’ psychological empowerment and school innovation climate on the relationship between inclusive leadership and innovative behavior among primary and junior teachers. A total of 358 valid questionnaires were administered to primary and junior teachers in mainland China using a convenience sampling approach. The data were analyzed using hierarchical linear modeling and bootstrap testing, revealing a positive relationship between principal inclusive leadership and teacher innovative behavior. Additionally, teachers’ psychological empowerment and school innovation climate were found to mediate this relationship, with the former playing a crucial role. The findings can enhance existing research on the impact of inclusive leadership in basic education. They also offer a new perspective for analyzing how inclusive leadership affects employees’ innovative behavior and provide valuable insights into stimulating innovation among primary and junior teachers.

## Introduction

The cultivation of innovative behavior is crucial for enhancing organizational efficiency and effectiveness in today’s rapidly evolving technological landscape and interconnected global communication^1^. In education, teacher innovative behavior is paramount as teachers serve as the backbone of the system and are indispensable in addressing challenges posed by rapid technological advancements and globalization^2,3^. Their ability to exhibit innovative behavior is vital in ensuring the system remains adaptable, dynamic, and responsive to current developments^4–6^. Therefore, fostering teacher innovative behavior becomes imperative for continuous progress in education.

Principal leadership is a crucial environmental factor that influences teacher innovative behavior^7^. Previous studies have investigated the impact of transformational leadership^8^, distributed leadership^9^, and empowering leadership^10,11^ on teacher innovative behavior. With the emergence of a new generation of teachers in mainland China, there is an increasing differentiation and diversification in their ideas, personalities, habits, and behaviors. Consequently, they expect schools to acknowledge and foster their uniqueness. This necessitates school leaders who can accommodate and support these diverse ideas and behaviors. However, more research is needed in the current literature regarding the influence of principal inclusive leadership on teacher innovative behavior, despite evidence demonstrating that inclusive leadership stimulates employee innovative behavior within the business sector^12,13^.

Previous studies have primarily examined the influence of inclusive leadership on employee innovation behavior through external organizational factors and internal psychological factors. From an external perspective, research has shown that inclusive leadership can stimulate innovative behavior by influencing the organization to support employees^14^, promoting knowledge sharing within the organization^15,16^, and fostering a climate of innovation^15^. From an internal perspective, studies have explored how inclusive leadership can enhance employee motivation^12,17^, psychological safety^18–21^, psychological empowermentt^22^, psychological capital^23,24^, and self-efficacy^24^ to promote innovative behavior. The two perspectives play a significant role in elucidating employee innovative behavior, yet they possess certain limitations: the external organizational culture perspective primarily emphasizes interpersonal interaction while overlooking individual factors; conversely, the inner individual psychological perspective tends to isolate individuals from groups and neglects the significance of group connection and support^25^. Therefore, it may be challenging to systematically understand the impact of inclusive leadership on employee innovative behaviors from a singular perspective, such as external organizational culture or internal individual psychology.

The Social Cognitive Theory (SCT) provides a comprehensive framework for understanding how behavior is influenced by personal, behavioral, and environmental factors^26^. In the educational context, SCT is particularly relevant for studying the relationship between leadership and teacher behavior. As an environmental factor, principal inclusive leadership indirectly influences teacher innovative behavior by shaping both the external school culture and the internal psychological states of teachers, aligning with SCT’s perspective on the dynamic interaction between environmental determinants and cognitive processes. Psychological empowerment, a pivotal cognitive mediator within SCT, is characterized by an individual belief in their capabilities to perform a task effectively. When school leaders exhibit inclusive behaviors, they can bolster teacher self-efficacy, thereby encouraging a greater propensity for innovation. Additionally, the school innovation climate, which is about the collective beliefs regarding the value and support for innovation within a school, directly affects teachers’ innovation motivation and behavior. At the same time, because of its inclusive characteristics, inclusive principals can easily form an innovative school climate.

Our research aims to comprehensively examine the intricate relationship between principal inclusive leadership and teacher innovative behavior, specifically investigating the mediating roles of teachers’ psychological empowerment and the school innovation climate. These mediating variables are fundamental components of Social Cognitive Theory (SCT), representing cognitive and environmental mechanisms through which leadership exerts influence. By integrating these concepts, our study provides a comprehensive understanding of how inclusive leadership can foster an environment conducive to teacher innovation, thereby contributing to the existing literature in this field. Furthermore, this study offers practical insights for educational administrators seeking to nurture innovative behaviors among teachers.

## Literature review and hypothesis development

### Principal inclusive leadership and teacher innovative behavior among primary and junior teachers

The impact of inclusive leadership on employees’ innovative behavior has been confirmed, and this leadership style can promote the entire process of employees' innovative behavior. Inclusive leadership engages employees in the decision-making process and encourages employees to speak openly^27^, which promotes the generation of new ideas^28^. Support from inclusive leaders in organizational resources facilitates the implementation of innovative ideas by employees^29^. Furthermore, inclusive leaders take responsibility for the ultimate outcomes of employee work, particularly when new ideas fail, thus encouraging employees to take more risks^30,31^. Literatures have also indicated that inclusive leadership can promote employee innovative behavior across different contexts^18,32,33^.

Although the impact of inclusive leadership on employee innovation in primary and junior education remains unexplored, we propose that inclusive principals enhance the social cognitive environment within schools by treating teachers equitably and valuing their opinions and ideas. This equitable treatment and value placement serve as vicarious experiences and verbal persuasions that can boost teacher self-efficacy, a crucial cognitive factor for fostering innovation. Inclusive principals cultivate a culture that aligns with Social Cognitive Theory's emphasis on creating a supportive environment to nurture innovation, encouraging teachers to engage in bold exploration and take responsibility for innovative endeavors. Inclusive leaders' tolerance for failure and psychological support creates a secure environment that motivates teachers to embrace innovative behaviors. Based on the aforementioned theories and empirical evidence, this study proposes the following hypotheses:

#### Hypothesis 1

Principal inclusive leadership positively impacts teacher innovative behavior.

### The mediating role of teachers’ psychological empowerment

Psychological empowerment reflects an individual response to the behavior of empowering parties and leaders, encompassing work meaning, self-efficacy, self-determination, and influence^34^. Previous studies have confirmed that psychological empowerment mediates between inclusive leadership and employee innovative behavior. According to Javed et al.^35^, employees perceive inclusive leadership as a supportive factor that enhances their psychological empowerment and stimulates innovative behavior.

From an educational perspective, inclusive leadership is a style that encourages principals to embrace teachers' diverse educational values, create empowering policies for both teachers and students, and prioritize educational justice and democracy^36^. This definition suggests that principal inclusive leadership involves authorization behaviors that foster psychological empowerment among teachers^37^. By fostering a work environment where teachers freely express their opinions, principals enable them to make value judgments about the purpose of their work based on their own beliefs and standards, thereby enhancing the meaning in their work. Furthermore, when principals reward teachers for successes and provide supportive encouragement during failures, they contribute to the development of teacher self-efficacy. The willingness of principals to support teachers in terms of resources, policies, and other aspects promotes their ability to control or make autonomous decisions regarding their work.

A substantial body of research has consistently demonstrated that when employees feel empowered, they exhibit higher proactivity and innovation^38^. Teachers within a school organization possess a rich cultural background and a strong desire for spiritual fulfillment. When the school provides them a sense of purpose, competence, autonomy, and influence in their work, they are more likely to reciprocate the support by implementing educational innovations.

According to social cognitive theory, self-concept serves as a mediating factor in the relationship between external social environmental factors and individual psychology and behavior. Self-concept encompasses an individual subjective perception of their physical state, personality traits, attitudes, social roles, and past experiences, as well as their attitudes, beliefs, and values^39^. In the context of education, teachers' psychological empowerment is a significant aspect of their work-related self-concept, highlighting their perceived capabilities, autonomy, and influence in their professional field. Leadership style is a crucial external factor that influences employees' psychological empowerment and subsequent behavioral manifestations, as established by prior research^40–42^. Building on this foundation, our study hypothesizes that principal inclusive leadership—characterized by openness, inclusiveness, and supportiveness—positively impacts teachers' psychological empowerment. By fostering empowerment, inclusive leaders initiate a chain of effects that ultimately stimulate and enhance teacher innovative behavior within the educational setting. Consequently, this study posits that principal inclusive leadership positively influences teachers' psychological empowerment and subsequently impacts their innovative behavior. In summary, the following hypotheses are formulated:

#### Hypothesis2

Teachers’ psychological empowerment mediates the relationship between principal inclusive leadership and teacher innovative behavior.

### The mediating role of school innovation climate

Ekvall believes that leadership behavior in the external environment significantly influences employees perception of organizational innovation climate^43^. Inclusive principals demonstrate openness by encouraging free discussions on educational matters and actively listening to teacher innovative ideas and solutions^44,45^, fostering a school environment that nurtures critical thinking and innovation. They also tolerate employee failures and mistakes^14^, creating a safe space for teachers to innovate and persist until they succeed^46,47^, inspiring other educators to participate in educational innovation through sharing diverse ideas and opinions, thus fostering a culture of courage and innovation within the school. Inclusive principals cultivate approachable relationships with teachers based on trust and respect, prioritizing the understanding of individual needs^48^ while providing essential resources for professional development. As a result, educators feel supported in cultivating an innovative climate.

Many studies have indicated that the school climate is crucial in shaping teacher innovative behavior. Such a climate can motivate teachers to learn new ideas and methods, leading them to change traditional teaching methods actively^49^. Schools that provide satisfaction, autonomy, and cooperation are more likely to have innovative teachers^50^. Additionally, Xia et al. has confirmed the relationship between innovation climate and teacher innovative behavior in mainland China higher education institutions. They concluded that a stronger school innovation climate encourages teachers to apply acquired knowledge and skills creatively in their work while solving tasks and problems with innovative approaches^51^. Similarly, Hou^52^ argues that the organizational climate of schools significantly influences the teaching innovation of primary and junior teachers.

Bandura's SCT emphasizes the role of observational learning, where individuals acquire new behaviors by observing others, particularly those in positions of authority or influence. In the context of educational institutions, principal inclusive leadership emerges as a critical environmental determinant shaping teacher behaviors and attitudes towards innovation. Specifically, the concept of reciprocal determinism within SCT underscores how individuals and their environments mutually influence each other. In this vein, principal inclusive leadership, characterized by active engagement, empowerment, and openness to diverse perspectives, constitutes a formidable environmental force that can foster a school culture conducive to innovation. As posited by Amabile et al.^53^, leaders play a pivotal role in either nurturing or suppressing subordinate innovative behaviors through the strategic construction of their organizational environment. Furthermore, the notion of vicarious experiences and self-efficacy embedded in SCT is pertinent to understanding how inclusive leadership can stimulate teacher innovation. As Yao^54^ illustrates, inclusive leadership fosters a team-level innovative climate by promoting a sense of belonging, encouraging idea sharing, and validating contributions from all team members. This climate acts as a catalyst for enhancing employees' belief in their ability to innovate—their self-efficacy—thereby stimulating innovative behavior. Accordingly, the following hypotheses are proposed in this paper:

#### Hypothesis H3

School innovation climate has a cross-level mediating effect between principal inclusive leadership and teacher innovative behavior.

### Comparison of double mediations

Drawing from the tenets of Social Cognitive Theory (SCT), which underscores the reciprocal interplay among personal factors, behaviors, and environmental influences, our study posits that teacher psychological empowerment and the school innovative climate function as critical mediators in translating inclusive leadership into teacher innovative behavior. However, the depth of this integration necessitates a closer examination through the lens of SCT's core principles, particularly the concepts of self-efficacy, observational learning, and reciprocal determinism.

In SCT, self-efficacy, a central component of psychological empowerment, is pivotal for innovation, as it refers to an individual belief in their ability to succeed in specific tasks. When principals empower teachers by granting autonomy over educational strategies, they nurture a sense of self-efficacy, personal meaning, and professional influence among teachers^55^. This aligns with SCT’s emphasis on vicarious experiences, where observing successful models (inclusive leaders) inspires similar behaviors. In this context, teachers perceive their role in school improvement as integral to their self-worth, fostering intrinsic motivation and a proactive stance towards innovation, even amidst challenges.

Contrasting this with the school innovative climate, an environmental factor under SCT, while essential for setting the stage for innovative behaviors, may not independently evoke the same level of engagement without the complementary subjective empowerment. The school objective environment, representing a facilitative condition for innovation, interacts with teacher subjective states in a reciprocal manner as per SCT’s reciprocal determinism. Yet, in instances where subjective empowerment prevails, it assumes a dominant role in shaping behavior, as personal beliefs and capabilities often dictate the threshold for action, especially in the face of adversity. The study suggests that the subjective "teacher psychological empowerment" pathway exerts a stronger impact on teacher innovation behavior than the objective "school innovation climate" pathway.

#### Hypothesis H4

The mediating effect of teachers' psychological empowerment is more vital than that of school innovation climate.

**Figure 1:**
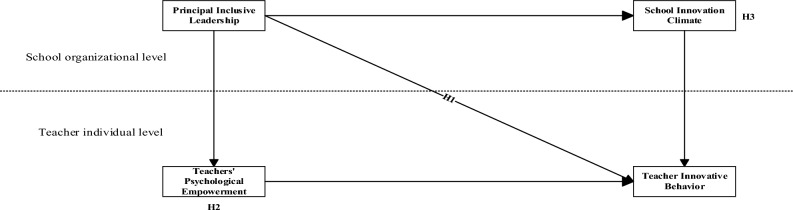
Research framework.

The research framework of this paper, based on the four research hypotheses, is shown in Fig. 1 below.

## Methodology

### Participants and procedure

In this study, we initially imported the prepared questionnaire into the Questionnaire Star platform to generate an electronic survey instrument. The electronic questionnaires were distributed to teachers currently employed in primary and junior schools in Guangdong Province, China, utilizing convenience sampling and snowball sampling methods through social platforms such as WeChat or QQ groups. Data collection took place from June 2023 to July 2023. 369 teachers from 42 primary and junior schools voluntarily and anonymously participated in this survey. After excluding invalid questionnaires containing missing or contradictory information, a final count of 358 valid questionnaires was obtained, resulting in an effective recovery rate of 97%. The demographic information of the valid sample is presented in Table 1.

**Table 1:** Respondent demographic data (N = 358).

Item	Category	Percentage	Item	Category	Percentage
Gender	Male	56.1	Academic title	Junior	40.3
	Female	43.9		Medium-grade	39.8
Age	≤ 25	19.0		Senior	19.9
	26–35	34.1	School location	Urban	52.9
	36–45	29.3		Rural	47.1
	≥ 46	17.6	Teaching experience (in years)	≤ 3	21.8
Level of education	College and below	31.6		4–10	37.1
	Undergraduate	57.5		11–20	28.1
	postgraduate	10.9		≥ 21	13.0

### Measures

To ensure our study's validity and cultural relevance, we employed established scales from western literature and meticulously translated them into Chinese through a rigorous two-step adaptation process. Firstly, bilingual experts proficient in both English and Chinese accurately captured each item original intent and meaning during translation. Subsequently, local education experts and practitioners reviewed the translated scales to ensure their suitability for our research context and participants. The content was assessed for its cultural appropriateness, relevance to the Chinese educational setting, as well as the target population comprehension level. Adjustments were made when necessary to reflect the unique nuances of the Chinese educational context while preserving the original theoretical foundations. All scale items were rated on a five-point Likert scale (ranging from 1 for strongly disagree to 5 for strongly agree), with higher scores indicating stronger agreement.

Principal inclusive leadership (PIL) is measured using a 9-item scale proposed by Carmelia et al.^48^, which mainly measures the three dimensions of openness, usability, and accessibility of principals. Examples of the items include: “My principal is willing to consider new ideas or suggestions from teachers” (openness), “I can always ask principal for advice if I have questions” (accessibility), and “I can find the principal to discuss new problems that arise in my job” (availability). The CFA indicated the good structural validity of the scale (X^2^/DF = 1.901, RMR = 0.018, RMSEA = 0.008, GFI = 0.990, IFI = 0.989, NFI = 0.991, TLI = 0.997, CFI = 0.989). The Cronbach’s α coefficient of this scale in this study was 0.871.

Teacher psychological empowerment (TPE) is measured using a 12-item scale proposed by Spreitzer^34^, which includes the four dimensions of competence, meaning, self-determination, and impact. Examples of the items include: "I feel that the work I do is very meaningful" (meaning), "I am confident that I have the basic ability to do a good job" (competence), "I can decide how to conduct my work" (self-determination),"I feel that I have a greater impact in my department" (impact). The CFA indicated the good structural validity of the scale (X^2^/DF = 2.153, RMR = 0.015, RMSEA = 0.019, GFI = 0.898, IFI = 0.974, NFI = 0.962, TLI = 0.886, CFI = 0.907). The Cronbach’s α coefficient of this scale in this study was 0.857.

Teacher innovative behavior (TIB) is measured using a 6-item scale proposed by Scott et al.^56^. Examples of the items include: “I search out new processes, techniques, and/or new ideas for my classes”, “I generate creative ideas”, “I promote and champions ideas to other teachers”, “I try to implement new ideas in the school”, “I develop adequate plans and schedules for the implementation of new ideas”. The CFA indicated the good structural validity of the scale (X^2^/DF = 1.796, RMR = 0.016, RMSEA = 0.015, GFI = 0.991, IFI = 0.977, NFI = 0.893, TLI = 0.978, CFI = 0.889). The Cronbach’s α coefficient of this scale in this study was 0.882.

The school innovation climate (SIC) is measured at the team level using the scale developed by Anderson et al.^57^, which includes four dimensions of vision, participation safety, task orientation, and innovation support. Examples of the items include: "Our teaching/grade subject group readily provides assistance for developing new ideas", and "Our teaching/grade subject group is dedicated to implementing innovative practices in their work." The CFA indicated the good structural validity of the scale (X^2^/DF = 1.792, RMR = 0.017, RMSEA = 0.016, GFI = 0.909, IFI = 0.987, NFI = 0.899, TLI = 0.974, CFI = 0.969). The Cronbach’s α coefficient of this scale in this study was 0.886.

### Data analysis

The cross-level dual-mediation model in this study was analyzed using SPSS22.0, Amos 21.0, and HLM6.0 software. The data analysis and processing steps were as follows: Firstly, the reliability and validity of four latent variables were examined using SPSS and AMOS software. Secondly, the aggregation effect of potential dimensions at the team level was assessed to determine if principal inclusive leadership and school innovation climate could be elevated to the team level based on meeting the standard of internal raters' reliability and within-group correlation coefficient. Then, inter-plane relationships (e.g. TPE to TIB, PIL to SIC) were tested with AMOS, while HLM was used for cross-layer inter-variable relationships (e.g. PIL to TIB, PIL to TPE, SIC to TIB). Finally, the mediation effect of hypotheses 2 and 3 was tested using the built-in Bootstrap program in AMOS. Additionally, to compare the influence of various mediators in this study involving dual mediation (hypotheses 4), we employed the program MacKinnon’s PRODCLIN2^58^.

### Ethics statement

This study adheres to the principles outlined in the Declaration of Helsinki and received approval from the Human Research Ethics Committee of Longgang Institute of Education Sciences, Shenzhen. All participants provided voluntary informed consent upon survey completion and withdraw from the study at any time. Furthermore, our data underwent anonymization procedures to ensure participant privacy.

## Results

### Reliability and validity test

The exploratory factor analysis was conducted for each potential variable, as presented in Table 2. The C.R. value exceeding 0.60 indicates good internal consistency of the variable. The AVE values for each latent variable in this model range from 0.458 to 0.785, demonstrating strong convergence validity for all four variables.

**Table 2:** Test table of mean value, standard deviation, correlation coefficient, reliability, and validity of each latent variable (N = 358).

Variable	M	SD	C.R	AVE	1	2	3	4
1. Principal inclusive leadership	3.432	0.801	0.912	0.689	**0.830**			
2. School innovation climate	4.001	0.526	0.854	0.458	0.385**	**0.677**		
3. Teachers’ psychological empowerment	4.208	0.638	0.802	0.626	0.419**	0.518**	**0.791**	
4. Teacher innovative behavior	3.789	0.512	0.925	0.785	0.588**	0.571**	0.468**	**0.886**

The diagonal bold data in the correlation matrix is the square root value of each variable AVE, and the lower triangle of the matrix is the Pearson correlation coefficient.

*M* is the mean value of the variable, *SD* is the standard deviation of the variable, *C.R.* is the constituent reliability, *AVE* is the average variance extracted amount.

### Descriptive statistics and correlation analysis

Correlation analysis was conducted on four variables: principal inclusive leadership, school innovation climate, teachers’ psychological empowerment, and teacher innovative behavior. The results are presented in Table 2. Principal inclusive leadership and teacher innovative behavior (r = 0.385, *p* < 0.01) showed a positive and significant correlation. Principal inclusive leadership positively and significantly correlated with school innovation climate (r = 0.419, *p* < 0.01). A positive and significant correlation existed between principal inclusive leadership and teacher psychological empowerment (r = 0.588, *p* < 0.01). Teacher innovative behavior exhibited a positive correlation with school innovation climate (r = 0.518, *p* < 0.01). Teacher psychological empowerment demonstrated a positive and significant correlation with teacher innovative behavior (r = 0.571, *p* < 0.01). School innovation climate displayed a positive correlation with teachers’ psychological empowerment (r = 0.468, *p* < 0.01). These results of the correlation analysis align with the expected correlations in the hypothesis model, providing foundational support for hypothesis testing.

If two variables have good discriminative validity, then the square root of the AVE of both variables should be higher than their correlation coefficients^59^. Table 2 shows the test of each variable differential validity. As can be seen from Table 2, the AVE square values of the four factors of the scale are all greater than their corresponding correlation coefficients, which proves that the scale has discriminative validity.

### Data aggregation check

The variables of principal inclusive leadership and school innovation climate are measured at the team level, requiring calculation of the reliability of rater R_wg_ within the group and the correlation coefficient ICC within the group. R_wg_ compares low-level variable variance with random distribution variance to determine if adding low-level variables to high-level ones is reasonable. ICC compares member variance within a group with mean between-group variance, including individual score confidence ICC (1) and average group score confidence ICC (2). In this study, the average R_wg_ for principal inclusive leadership is 0.918 (> 0.7), ICC (1) value is 0.685(> 0.12), and ICC (2) value is 0.939 (> 0.6). For school innovation climate, the average R_wg_ is 0.879 (> 0.7), ICC (1) value is 0.607 (> 0.12), and ICC (2) value is 0.818 (> 0 0.6). Both dimensions meet the criteria. Thus, the two variables of principal inclusive leadership and school innovation climate can be extended from individual measurement scores to the group level.

### Model fit test

The model fitting indexes in this study (X^2^/DF = 2.701, RMSEA = 0.063, GFI = 0.908, AGFI = 0.910, CFI = 0.901) indicate a strong fit for the proposed model according to Schreiber et al.^60^ criteria, suggesting its suitability for path coefficient analysis and hypothesis testing.

### Direct effect hypothesis testing

The primary hypothesis of this study is limited to hypothesis 1, which examines the direct impact of PTL on TIB. This study also investigates the effects of other variables to comprehensively comprehend the relationship between each variable and adequately prepare for subsequent mediation hypothesis testing. The analysis results pertaining to the influence relationship between pairs of variables are presented in Table 3. The findings indicate that principal inclusive leadership significantly impacts school innovation climate, and teachers’ psychological empowerment significantly influences teacher innovation behavior (*p* < 0.01). Because the impact of PIL on TIB (H1), SIA on TIB, and PIL on TPE are cross-level effects, these tests were analyzed using HLM software. Here are the specific steps: Firstly, the group mean value of layer 1 is centralized, and then the zero model is tested. The intra-group correlation coefficients ICC (1) for calculating teacher innovation behavior and teachers’ psychological empowerment are 0.279 and 0.248, respectively, greater than 0.06. Therefore, it is necessary to conduct a multi-level analysis and establish a multi-level regression model for hypothesis testing. Through the results, it can be found that the influence relationship between H1 and other variables has passed the test.

**Table 3:** Results of hypothesis testing.

Level	Model	Estimation of parameters			
		Standardized estimates	S. E	C.R.(T)	*p*
Same level	PIL → SIA	0.449	0.027	10.918	***
	TPE → TIB	0.508	0.059	10.239	***

Level	Model	Estimation of parameters			
		γ_00_	γ_01_	σ^2^	τ_00_
Cross-level	Null model	5.128***		0.679	0.269***
	PIL → TIB (H1)	4.268***	0.419***	0.501	0.109***
	SIA → TIB	4.368***	0.323***	0.571	0.068***
	Null model	5.338***		0.719	0.239***
	PIL → TPE	4.489***	0.489***	0.558	0.061***

***means *p* < 0.001, γ_00_ means the intercept term, γ_01_ means the path regression coefficient, σ^2^ means the residual of layer 1, and τ_00_ means the residual of the intercept, S. E means standard error.

### Mediating effect hypothesis testing

As for the mediating effect test, the first method is the causal step method proposed by Kenny and Berron, which tends to underestimate the statistical error I rate. Sobel then developed the product of the coefficient’s method. This simple Z-value test applies only to a single intermediate variable and requires a normal distribution of data for coefficient products. In recent years, Mackinnon proposed the Bootstrap confidence interval method, which does not require normality assumption in sampling distribution and can test multiple intermediary variables applicable to small and medium-sized samples. Since this study aims to test double cross-level mediation effects, we adopt this method. In order to make the results more reliable, the coefficient product Z is used for auxiliary verification. The specific steps are as follows:

The AMOS21.0 Bootstrap function was applied, the sample number of Bootstrap was selected as 5000, the confidence interval was selected as 95%, and the maximum likelihood method was applied to perform the calculation. The Bootstrap confidence interval method is divided into two interval estimation methods: the "Bias-Corrected" method and the "Percentile" method. The total effect, indirect effect, and direct effect confidence interval estimation results obtained by the two methods on principal inclusive leadership on teacher innovative behavior are shown in Table 4.

**Table 4:** Confidence interval estimation (N = 358).

Model	Estimated value	Product of coefficients		Bootstrapping (5000 times)			
		S.E	Z	Lower (Bias)	Upper (Bias)	Lower (Percentile)	Upper (Percentile)
Total effect							
PIL → TIB	0.309	0.026	11.298	0.259	0.369	0.261	0.373
Indirect effect							
PIL → TIB	0.351	0.031	10.139	0.289	0.428	0.279	0.419
Direct effect							
PIL → TIB	0.079	0.073	2.398	0.012	0.142	0.007	0.141

The Bias-Corrected and Percentile 95% confidence interval of the total impact of principal inclusive leadership on teacher innovative behavior are 0.259–0.369 and 0.261–0.373, both excluding 0, and the Z-value of 11.298 is greater than 1.96. Therefore, the mediation effect may exist and can be tested in the next step. The Bias-Corrected and Percentile 95% confidence intervals of the indirect impact of principal inclusive leadership on teacher innovative behavior are 0. 289–0. 428 and 0. 279–0. 419, both excluding 0, and the Z-value of 10.139 is greater than 1.96. So mediation effect exists. Moreover, the bias-corrected and Percentile 95% confidence intervals of principal inclusive leadership on teacher innovative behavior are 0.012–0.142 and 0.007–0.141, both of which exclude 0. In addition, the Z-value of 2.398 is higher than 1.96, so it is believed that the direct influence of principal inclusive leadership on teacher innovative behavior exists. Therefore, the mediating effect of principal inclusive leadership on teacher innovative behavior belongs to a partial mediating effect.

Although indirect effects have been verified, this is only the total indirect effect between the two variables, which can only indicate that at least one mediated influence path exists. However, further research is needed to determine which specific mediating effect exists and the relative importance of these two mediating effects in the overall indirect effect. Confidence intervals were calculated using PRODCLIN2 software developed by Mackinnon to assess the strength of different mediation paths. Table 5 shows that both mediation paths have 95% confidence intervals that do not contain zero, indicating that they exist and that the tests for hypothesis 2 and 3 pass. Furthermore, the "principal inclusive leadership → teachers' psychological empowerment → teacher innovative behavior" path has most significant influence (accounting for 54.20% of the total indirect effect), highlighting psychological empowerment as a critical mediator between principal inclusive leadership and teacher innovative behavior.

**Table 5:** Effect test of mediation path (N = 358).

Mediation path	Mackinnon PRODLIN2 95% CI		Indirect standard	Impact strength (%)
	Lower	Upper		
PIL → TPE → TIB	0.123	0.226	0.411	54.20
PIL → SIA → TIB	0.062	0.140		30.30

## Discussion

### The effect of principal inclusive leadership on teacher innovative behavior

The study found that principal inclusive leadership independently predicted teacher innovative behavior; the stronger the principal inclusive leadership is, the more teacher innovative behaviors can be stimulated. This observation is consistent with previously published literature, which demonstrated associations between inclusive leadership and innovative behaviors in Western^22^ and Asian societies^61^. Using snowball sampling, Zhong et al.^62^ surveyed 523 individuals and 74 leaders from 55 organizations in Shanghai and Shandong Province, China, finding a positive correlation between inclusive leadership of supervisors/managers and innovative behavior among frontline employees. This study further validates and expands research on the influence of inclusive leadership on employee innovation within the context of primary and junior education in China. The principal inclusive leadership significantly predicts teacher innovative behavior, but the specific underlying mechanisms linking this meaningful relationship remain unclear.

### The mediating effects of teachers' psychological empowerment and school innovation climate

Our findings demonstrate that the relationship between principal inclusive leadership and teacher innovative behavior is mediated by two pathways, namely teachers’ psychological empowerment and school innovation climate. This is the first study to disentangle the complex interrelationships among principal inclusive leadership, teacher innovative behavior, teachers’ psychological empowerment, and school innovation climate, particularly in low- and middle-income countries. Our research advances our understanding of the underlying mechanisms linking inclusive leadership with innovative behavior.

This study has demonstrated that teachers' psychological empowerment mediated the relationship between principal inclusive leadership and teacher innovative behavior, explaining 54.2% of the overall indirect effect. This observation fortified our fourth hypothesis that principal inclusive leadership was more likely to stimulate teacher innovative behavior via teachers' psychological empowerment. This finding is consistent with previous research^63^ and can be explained by self-determination theory^64^. Javed et al.^30^ found that inclusive leadership, as a supportive external context, has been found to meet individual basic psychological needs for competence, autonomy, and relatedness, thereby enhancing their motivation to engage in innovative behavior. In high-power distance contexts such as China, leadership behaviors and attitudes significantly impact subordinate affective states, cognitive processes, and work-related behaviors. Specifically, primary and junior principals who are open, available, and accessible can positively influence teachers' perceptions of their competence, autonomy, and the meaningfulness of their work. As a result, this approach can enhance teacher perceived psychological empowerment, leading to increased autonomy, efficacy, and meaning in their work. This enables them to identify problems promptly and offer innovative suggestions.

The present study further reveals that the school innovation climate serves as a mediator in the association between principal inclusive leadership and teacher innovative behavior, accounting for 30.3% of the overall indirect impact. Although no literature directly proves the link between principal inclusive leadership mediated by school innovation climate and teacher innovative behavior, existing similar research consistently shows that the innovation climate acts as a mediator between various leadership styles and employees' innovative behavior. For instance, Hou (2018) found that the school innovation climate mediates the influence of transformational and transactional leadership on teacher innovative behavior^52^, while Sagnak (2012) confirmed that empowering leadership impact on teacher innovative behavior is mediated by the school innovation climate^65^.

### Differential mediation effects: innovation climate versus psychological empowerment

The finding of this study, that teachers' psychological empowerment has a greater impact on their innovative behavior than the school innovation climate, aligns perfectly with SCT. SCT emphasizes the reciprocal interaction of personal factors, environmental influences, and behavior, highlighting the importance of cognitive processes and self-efficacy in shaping actions. In this context, teachers' psychological empowerment acts as a strong internal motivator, reflecting their subjective evaluation of the significance, ability, autonomy, and influence of their work^34^. The school climate of innovation, which is influenced by the inclusive leadership of the principal and fosters a supportive context for innovation, plays a secondary role compared to internal empowerment. This finding emphasizes SCT's proposition that although environmental conditions, such as a positive innovation climate, provide opportunities and reinforcement, it is the internalization of these external cues through personal beliefs and efficacy expectations that activate and sustain innovative behaviors^26^. Teachers who experience heightened psychological empowerment, demonstrate a stronger emotional attachment to their organization^66^, and are motivated to reciprocate support from inclusive leaders through citizenship behaviors^67^.

By reframing the discourse within the framework of SCT, our research highlights the significance of fostering individual belief systems and personal empowerment strategies in conjunction with cultivating supportive organizational environments. This amplifies SCT's explanatory capacity and practical implications for educational leadership and policy.

### Practical implications

First, our research has shown that principal inclusive leadership positively impacts teacher innovative behavior. To foster such behavior, educational administration could appoint principals who possess qualities like openness, availability, and accessibility. Moreover, training programs for primary and junior school principals could incorporate relevant theories on inclusive leadership to help them comprehend the significance of embracing diverse educational perspectives from teachers, tolerating mistakes and failures during educational reforms, and actively supporting teachers' exploration of teaching methods.

Second, the study underscores the pivotal role of psychological empowerment in stimulating teacher innovative behavior, a role that is more substantial than the influence of the school innovation climate. This suggests that internal motivational factors, such as teacher self-efficacy, autonomy, and perceived influence, are paramount in driving innovation. Therefore, it is crucial for school leaders to prioritize strategies that amplify teachers' psychological empowerment to cultivate innovation. Principals can actively engage teachers in meaningful organizational decisions, ensuring their perspectives are valued and integrated into school policies and practices. By doing so, leaders can create an environment that recognizes teachers' contributions and instills a sense of ownership and self-determination. Providing targeted support, professional development opportunities, and a culture of respect can significantly bolster teachers' psychological well-being and their belief in their capacity to innovate.

Finally, the present study found that a positive school innovation climate promotes teacher innovative behavior, effectively fostering creative teaching. Hence, it is imperative for school administrators to strategically cultivate a shared perception among teachers regarding the supportive climate for educational and pedagogical innovation within their institutions. This will enable all educators to acknowledge and appreciate the paramount importance placed on innovation by school leaders. Moreover, administrators could offer timely assistance and support to teachers encountering challenges during the implementation of innovative approaches. Furthermore, they could exhibit utmost tolerance towards setbacks or struggles encountered while innovating, while simultaneously recognizing and rewarding those who achieve success through educational innovations.

### Limitations

Although this study reveals certain insights, some limitations should also be taken into consideration. First, although various means have been used to avoid subjectivity and social expectation effects, the objectivity of the results will still be questioned to some extent. In the future, behavioral experiment method can be used as the aid to design special situations, select appropriate subjects, and combine questionnaire surveys to obtain more objective research results. Second, sample data are mainly analyzed using cross-sectional data, which cannot explain the causal relationship between variables well. In future studies, longitudinal investigation method of interval time is considered to further verify the causal relationship between variables. In addition, it is important to note that our samples are exclusively from Guangdong Province, Chin, which raises concerns about the generalizability of our findings. While the surveyed city currently represents other similar cities in China, it is crucial to acknowledge potential cultural, institutional, or societal variations when attempting to generalize these results internationally. To address this concern in future studies, expanding the survey scope and increasing the sample size should be considered for the robustness and applicability of research conclusions.

## Conclusions

Based on the social cognitive theory, this study investigates the impact of principal inclusive leadership on innovative behavior among primary and junior teachers, as well as the underlying mechanisms. An analysis of 358 questionnaires collected from primary and junior teachers reveals a positive association between principal inclusive leadership and the innovative behavior of primary and junior teachers. The mediating effects of teachers' psychological empowerment and school innovation climate play a crucial role in explaining the relationship between inclusive leadership and teacher innovative behavior. Specifically, it is observed that the mediating effect of teachers' psychological empowerment is stronger than that of school innovation climate in linking principal inclusive leadership with teacher innovative behavior.

## Data Availability

For inquiries regarding the data and materials in this paper, please contact the corresponding authors at billbao@126.com.
